# Identification and Antithrombotic Activity of Peptides from Blue Mussel (*Mytilus edulis*) Protein

**DOI:** 10.3390/ijms19010138

**Published:** 2018-01-04

**Authors:** Meiling Qiao, Maolin Tu, Zhenyu Wang, Fengjiao Mao, Hui Chen, Lei Qin, Ming Du

**Affiliations:** 1 School of Food Science and Technology, Dalian Polytechnic University, National Engineering Research Center of Seafood, Dalian 116034, China; 15108320017227@dlpu.edu.cn (M.Q.); wangzhenyu@dlpu.edu.cn (Z.W.); 15108320017201@dlpu.edu.cn (F.M.); 16308320017008@dlpu.edu.cn (H.C.); qinlei@dlpu.edu.cn (L.Q.); 2 Department of Food Science and Engineering, Harbin Institute of Technology, Harbin 150090, China; 15B925042@hit.edu.cn

**Keywords:** *Mytilus edulis*, protein, enzymatic hydrolysis, UPLC-Q-TOF-MS/MS, in silico analysis

## Abstract

The blue mussel (*Mytilus edulis*) reportedly contains many bioactive components of nutritional value. Water-, salt- and acid-soluble *M. edulis* protein fractions were obtained and the proteins were trypsinized. The resultant peptides were analyzed by ultra-performance liquid chromatography quadrupole time of flight tandem mass spectrometry (UPLC-Q-TOF-MS/MS). 387 unique peptides were identified that matched 81 precursor proteins. Molecular mass distributions of the proteins and peptides were analyzed by sodium dodecyl sulfate-polyacryl amide gel electrophoresis (SDS-PAGE). The differences between the three protein samples were studied by Venn diagram of peptide and protein compositions. Toxicity, allergic and antithrombotic activity of peptides was predicted using database website and molecular docking respectively. The antithrombotic activity of enzymatic hydrolysate from water-, salt- and acid-soluble *M. edulis* protein were 40.17%, 85.74%, 82.00% at 5 mg/mL, respectively. Active mechanism of antithrombotic peptide (ELEDSLDSER) was also research about amino acid binding sites and interaction, simultaneously.

## 1. Introduction 

*Mytilus edulis* (*M. edulis*) is a typical marine bivalve mollusk dwelling on beach rocks. It reportedly contains many types of bioactive components of nutritional value with pharmaceutical activities, e.g., nourishing the liver and kidneys, adjusting the blood pressure, curing night sweats, dizziness, impotence, etc. [1,2]. *M. edulis* is widely artificially cultivated and also globally popular with customers. However, the vast majority are freshly consumed, which leads to their low economic value and waste of the protein resources.

Based on the differences in solubility, the mussel protein is usually divided into a water-soluble protein fraction (sarcoplasmic protein), salt-soluble protein fraction (myofibrillar protein) [3] and water-insoluble protein fraction (matrix protein) [4,5]. At present, shellfish protein can be a natural source of bioactive peptides like milk proteins [6], peptides are very important in various food product applications.

On the other hand, bioactive peptides may be used in the treatment of the human disease and attract increasing attention because of their beneficial effects and better stability than proteins. Many studies showed that intact proteins are difficult to digest and absorb after intake by the human body because of their large molecular mass and complicated three-dimensional structure; these features significantly affect their physiological function and nutritional value [7]. Since peptide structure is simple and the molecular mass is much smaller than protein, peptides are well known as bioactive substances involved in various cellular functions in the human body. In addition, composition of allergenic food protein, e.g., wheat gluten [8], caseins [9] and β-lactoglobulin [10], are less allergic. The composition of shellfish may be developed as seafood seasoning, health food, nutritional supplement, natural medicine and protein additives in food products. These peptides may, e.g., regulate gastrointestinal motility and the immune system [11]. They possess anti-bacterial properties [12], converting enzyme inhibitory activity [13] as well as anti-cancer, anti-oxidation [14,15] and antihypertensive activity [16].

The aim of this study was to extract *M. edulis* proteins based on their solubility. Enzymatic hydrolysates of *M. edulis* were prepared from three different-solubility protein fractions and peptides in the hydrolysates were identified by ultra-performance liquid chromatography quadrupole time of flight tandem mass spectrometry (UPLC-Q-TOF-MS/MS). Moreover, allergic, toxicity and antithrombotic activity were predicted in silico, respectively. The antithrombotic activity and active mechanism of antithrombotic peptide were determined. This study comprised a preliminary analysis of these peptides, paving the way for a better understanding of bioactive peptide and protein.

## 2. Results and Discussion

### 2.1. Identification and Characterization of M. edulis Proteins and Identification of Peptides by Enzymatic Hydrolysis

The three protein fractions, P1–P3, isolated from the mussel represented 48.40 ± 2.76% (*w*/*w*), 17.19 ± 0% and 11.39 ± 0.64% (*n* = 6) of the total protein, respectively. The total protein extraction rate was 76.98 ± 3.40%. The water-soluble protein fraction (P1) was soluble both in water and a diluted salt solution, while the salt-soluble protein fraction (P2) could only be dissolved in a high-salt solution. The water-insoluble protein could be extracted with an acid or alkali. On the other hand, the protein could be also simply classed as cellular protein and extracellular protein [4,5].

**Figure 1:**
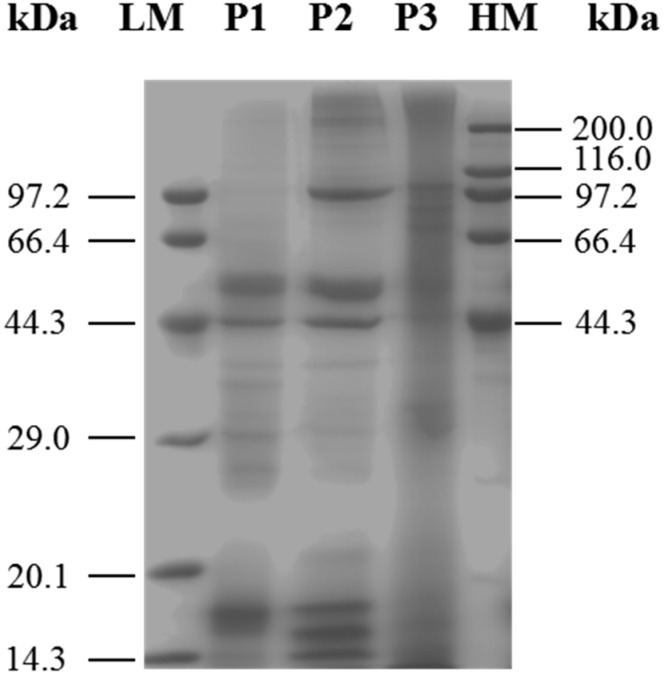
SDS-PAGE analysis of different-solubility protein fractions from *M. edulis was shown*. P1: proteins extracted in 0.05 M PBS (pH 7); P2: proteins extracted in 0.1 M PBS (pH 7) containing 0.6 M NaCl; P3: proteins extracted in 3% (*w*/*v*) citric acid solution, with heating. LM: Premixed Protein Marker (Low, 14.3–97.2 kDa); HM: Premixed Protein Marker (High, 44.3–200.0 kDa).

SDS-PAGE is widely used for the isolation and identification of mixed proteins [17]. After vacuum freeze-drying, the three protein fractions were analyzed by SDS-PAGE (Figure 1). The results indicated that although these three protein fractions shared proteins of similar molecular weight, each of them also contained unique proteins. Each sample was used. Lane 1: marker; lane 2: P1; lane 3: P2; lane 4: P3. Characteristic bands of 29.51, 39.98, 44.92 and 53.67 kDa were shown in lane 2; 17,47, 44.71, 52.13, 99.7 and 215 kDa band were particularly observed in lane 3, protein bands at 75.33, 88.55, 106.69 and 177.33 kDa were identified in lane 4.

**Figure 2:**
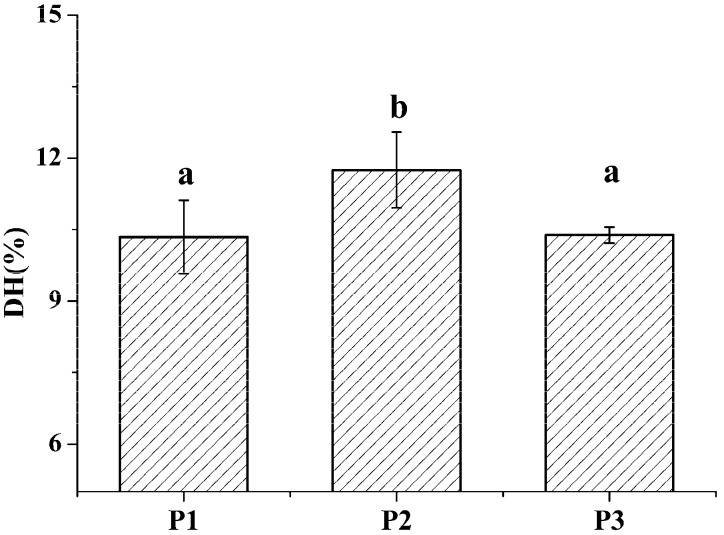
The degree of hydrolysis of *M. edulis* proteins from different fractions was shown. Values designated by different letters (a,b) are considered statistically different (*p* < 0.05). All statistical tests (*n* = 3) were conducted using SPSS software 19.0 (SPSS Inc., Chicago, IL, USA).

**Figure 3:**
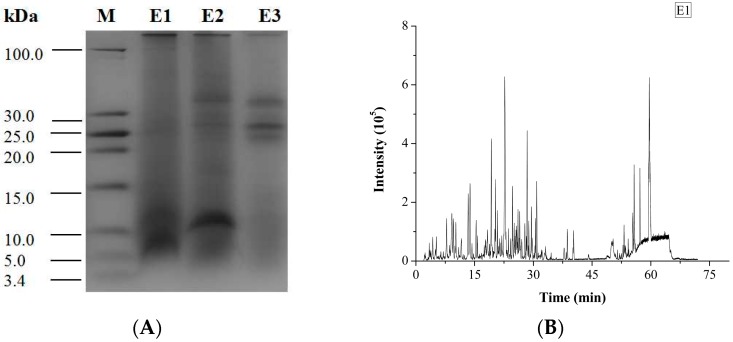

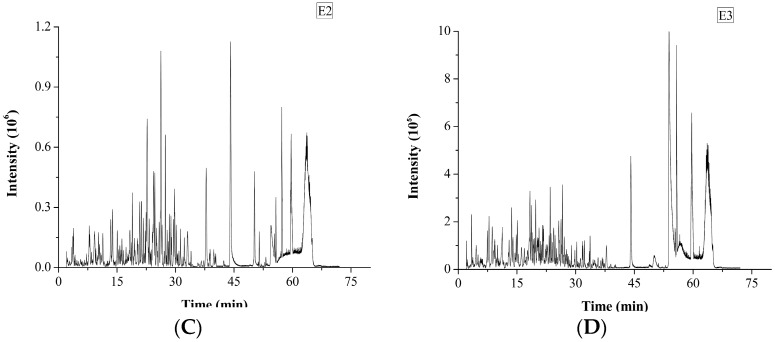
Tricine-SDS-PAGE analysis and Base Peak Chromatogram spectrum of enzymatic hydrolysates of *M. edulis* protein fractions were shown. (**A**) The peptides were electrophoresed on 4% stacking gel and 16% separating gel. (**B**–**D**) Enzymatic hydrolysis products in samples E1, E2 and E3 correspond to the hydrolysates of P1–P3 respectively and base Peak Chromatogram spectrum of enzymatic hydrolysates of *M. edulis* protein fractions.

The degree of hydrolysis (DH) of different protein fractions was determined (Figure 2). Although the hydrolysis was carried out under similar conditions, some pronounced differences were observed between the samples. The DH of the salt-soluble protein fraction was significantly higher (*p* < 0.05) than the DH of the other two protein fractions. At the same time, there was no significant difference (*p* < 0.05) in the DH value of the two latter protein fractions. Tricine-SDS-PAGE analysis revealed that the enzymatic hydrolysate of the water-soluble protein fraction mainly consisted of 10.00 kDa peptide, while the enzymatic hydrolysate of the salt-soluble protein fraction contained a wide variety of peptides with masses from 7 to 40 kDa (Figure 3A). Further, the enzymatic hydrolysate of the P3 fraction contained peptides with masses of 30 kDa. The distribution of molecular masses of peptides in the enzymatic hydrolysates was consistent with the distribution of proteins fractions. Base Peak Chromatogram spectrum of hydrolyzed fractions was shown in Figure 3B, which indicated that peptides were well isolated. Peptides in enzymatic hydrolysates of fractions P1–P3 were analyzed; 387 different peptides were identified by UPLC-Q-TOF.

### 2.2. In Silico Prediction of Peptides from M. edulis

**Figure 4:**
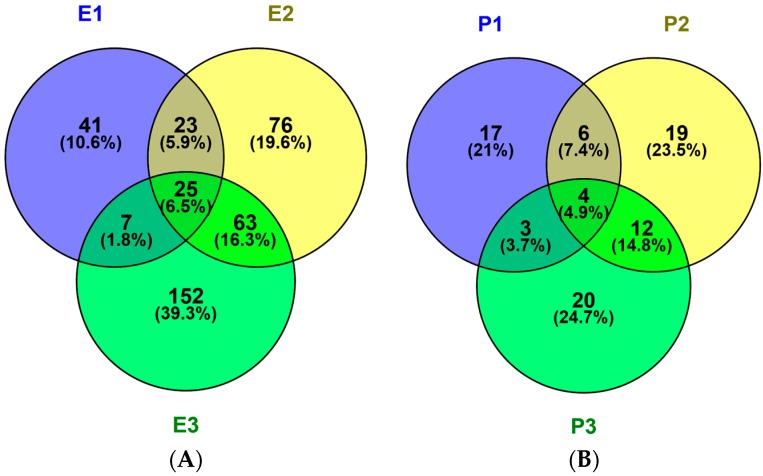
Venn diagrams of peptides (**A**) and proteins (**B**) in hydrolysates of different protein fractions from *M. edulis*. 387 different polypeptides and 81 proteins were identified in the sarcoplasmic (P1), myofibrillar (P2) and matrix (P3) protein fractions. Venn diagrams were painted with Venny 2.1.0. Enzymatic hydrolysis products named E1, E2 and E3.

The potential toxicity and allergic of all peptides was assessed using ToxinPred and AlgPred. All peptides show no evidence of toxicity and allergic in silico (Table 1, –CDOCKER ENERGY > 150). The numbers of unique peptides identified in the E1, E2 and E3 fractions were 96,187 and 247, respectively and their activities, toxicities and allergic were predicted. The sequences, peptide sources, toxicities and allergic of peptides with –CDOCKER ENERGY score >150 were listed in Table 1. The activity became stranger as the score become larger. The number of peptides with scores more than 150 were 10, 18 and 19 identified in P1, P2 and P3, respectively. Peptides information was summarized in Figure 4A. Fractions E1, E2 and E3 contained 41, 76 and 152 unique peptides, respectively, 25 peptides were shared. So, the antithrombotic activity of E2 and E3 was different from E1 because of number and type about peptides.

The possible protein sources of peptides were also identified. In the three protein fractions (P1, P2 and P3), 30, 41 and 39 proteins were identified, respectively in Figure 4B. Thirty *M. edulis* proteins were identified, including paramyosin, tropomyosin, collagen-like protein, filamin-like protein, histone, transgelin-like protein, etc. Proteins similar to proteins from other shellfish such as *Crassostrea gigas*, *Pinctada fucata* and *Crassostrea angulata*, were also identified. The sequence coverage for tropomyosin in the three samples, P1, P2 and P3, was 37%, 46.8% and 26.4%, respectively. More tropomyosin had been identified in P2 contrast to P1 and P3 due to its salt-soluble property. More and more proteins will be identified from *M. edulis* with the development of MS technology in the future.

### 2.3. Antithrombotic Activity of E1–E3 and Active Mechanism of Antithrombotic Peptide

**Figure 5:**
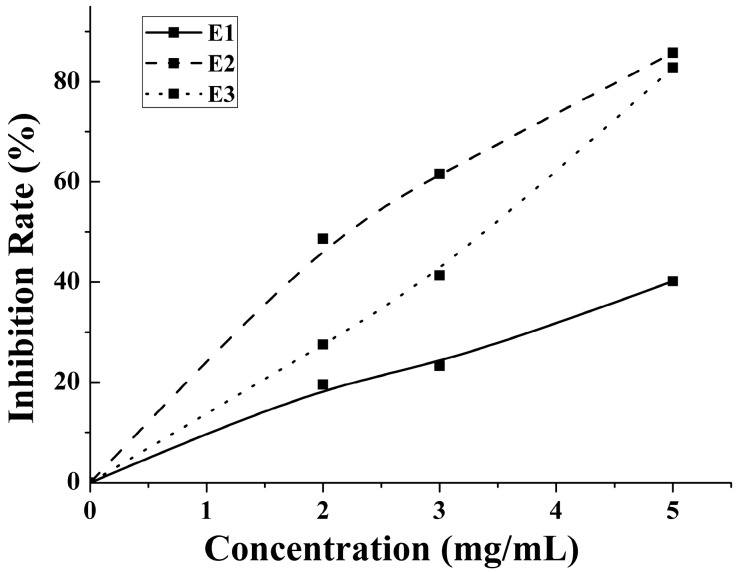
The anticoagulant activity of 2, 3 and 5 mg/mL E1–E3 were determined.

An enzymatic hydrolysis approach was proposed for the preparation of bioactive hydrolysate of *M. edulis* protein. The inhibition rate increased with the concentration increases. The antithrombotic activity of enzymatic hydrolysates from water-, salt- and acid-soluble *M. edulis* protein were 40.17%, 85.74%, 82.00% at 5 mg/mL, respectively. The inhibition rate of E2 was significantly higher than E1 and similar with E3 in Figure 5. On the one hand, the DH of P2 fraction was significantly higher (*p* < 0.05) than the DH of the other two protein fractions. On the other hand, the number and type of peptides from E2 and E3 were more than E1. Peptide distributions of E2 and E3 were similar in Figure 4A. In total, results showed that E3 and E2 had better activity than E1 and the antithrombotic activity of hydrolysates may be related to protein type, the DH and types and properties of peptides according to active mechanism.

There were many ways to inhibit thrombin; on the one hand, a stable thrombin-antithrombin complex formed by peptide and active sites inhibited activity of thrombin, and fibrinogen was transformed into fiber protein in order to achieve the antithrombotic activity. On the other hand, thrombin played an important role in the hydrolysis process, the enzyme cutting site of thrombin was in the middle of RG from peptide, which slowed down the process of thrombin and fibrinogen [18,19]. Peptides containing an RG structure were competitive with fibrinogen. RGILTLK, RGMVAGDSK and RGVNDELVY were hydrolyzed by thrombin which identified from E2 and E3, separately, which may also lead to higher activity.

**Figure 6:**
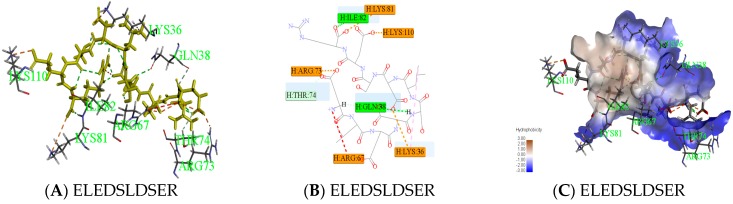

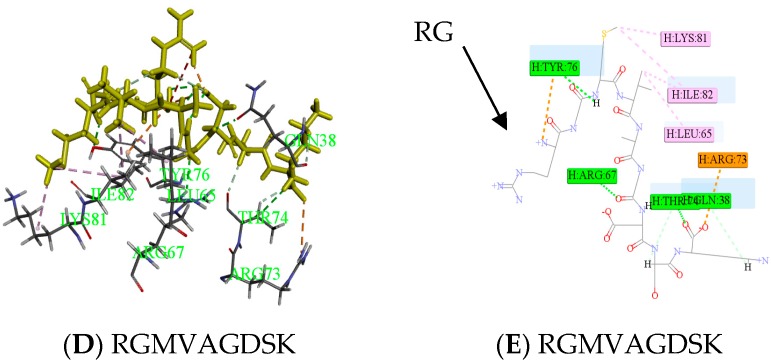
Active mechanism of peptide and amino acids of thrombin interact with peptides (ELEDSLDSER and RGMVAGDSK) were shown. Yellow sticks were peptides and others were amino acids identified by the green label in (**A**,**D**). All interactions between peptides and thrombin were shown in (**B**,**E**). Different colors represented different roles. The hydrophobic interaction was done in (**C**). The closer the color gets to darker blue, the stronger the hydrophilic.

Peptides (ELEDSLDSER) appeared in result of molecular docking might be more active. Docking for the interaction of peptides with thrombin was shown in Figure 6A. Amino acids which combined from thrombin of ELEDSLDSER were Lys36-Gln38-Arg67-Arg73-Thr74 -Lys81-Ile82-Lys110. Amino acids that have hydrogen bonding were Gln38 and Ile82, the number of hydrogen bonds were 1and 1, respectively. There was a carbon hydrogen bond in Thr74, many salt bridges and attractive charges in other amino acids in Figure 6B, which made the compound more stable. So, the score of ELEDSLDSER was highest. At the same time, Gln38 and Thr74 were reported as key binding sites [20,21]. Two parts had strong hydrophilic shown in Figure 6C, which may be conducive to structural stability. Meanwhile, Peptide RGMVAGDSK came from E2, was shown in Figure 6. The amino acid combined with thrombin was Gln38-Leu65-Arg67-Arg73-Thr74 –Tyr76-Lys81-Ile82 in Figure 6D, it was worth that hydrolysis position of RG was exposed and there was no interaction between them in Figure 6E.

## 3. Materials and Methods

### 3.1. Materials and Chemicals

*M. edulis* was purchased in Changxing market of Dalian. Trypsin (EC3.4.21.5, 2.5 × 10^5^ U/g) was purchased from Solarbio (Beijing, China). Formic acid (FA) and acetonitrile (High Performance Liquid Chromatography grade) were supplied by Sigma-Aldrich Co. (St. Louis, MO, USA). Cleanert C18-N solid phase extraction (SPE) column was purchased from Beijing Baoxidi Science and Technology Co., Ltd. Fibrinogen and thrombin were purchased from Sigma-Aldrich Co. (St. Louis, MO, USA). All other chemicals used in this study were analytical grade.

### 3.2. Preparation of M. edulis Proteins and Enzymatic Hydrolysis

Fresh mussels were washed and removed the shell and the byssus. Mussels were sheared and starched in 8000× *g* (IKA, Staufen, Germany). Proteins were extracted from fresh *M. edulis* specimens (*n* = 3) according to their solubility. Briefly, the extraction procedure was as follows. First, the mussel meat slurry was suspended (1:6 *w*/*v*) in 0.05 M phosphate butter solution (pH 7) and incubated for 60 min at 4 °C to extract the first proteins fraction (P1); the samples were then centrifuged (4 °C, 10,000× *g*, 15 min) and the supernatant was collected. The pellet was then suspended (1:9 *w*/*v*) in 0.1 M phosphate butter (pH 7) containing 0.6 M sodium chloride and incubated for 30 min at 4 °C to extract the second proteins fraction (P2); Protein solution was centrifuged and the supernatant collected. Finally, the third protein fraction (P3) was extracted from the pellet from the second centrifugation by incubating (1:15 *w*/*v*) in 3% (*w*/*v*) citric acid solution at 70 °C in a water-bath. Similarly, this solution was also centrifuged and the supernatant collected. The salt was removed from protein extracts by dialysis with a membrane with cut-off of 3000 Da. The three protein fractions were dialyzed and vacuum freeze-dried (Ningbo ShuangJia Instrument Co., Ltd., Ningbo, China). The protein content in samples was determined by the Kjeldahl method [22,23].

Protein fractions were diluted 2% (*w*/*v*) in water and trypsinized (5000 U/g, 45 °C, pH 8.5) because of the ubiquity and controllability of trypsin [24,25]. The final degree of hydrolysis was determined at 3 h, because DH gradually increases before 3 h and there was no significant change after that. DH value was measured using the “pH-state” method as follows:

(1) $$\mathrm{Degree}\;\mathrm{of}\;\mathrm{hydrolysis}(\mathrm{DH},\%)=B\times \mathrm{Nb}\times \frac{1}{\alpha }\times \frac{1}{MP}\times \frac{1}{h_{tot}}\times 100\%$$

where B is the volume of consumed NaOH solution (mL), Nb is the concentration of NaOH solution, *α* is the degree of dissociation of α-amino acid, α = [10(pH − pK)]/[1 + 10(pH − pK)] (pH = 8.5, pK = 7.5). *MP* is the total mass of the substrate protein (g) and *h_tot_* is the total quantity of peptide linkage unit of substrate protein (mmol/g). Three different peptide samples were named E1, E2 and E3 and the hydrolysates of them corresponded to P1, P2 and P3, respectively.

### 3.3. Sodium Dodecyl Sulfate-Polyacryl Amide Gel Electrophoresis (SDS-PAGE)

SDS-PAGE was performed using AE-8135 (ATTO Corporation, Tokyo, Japan). Premixed Protein Marker (Low, 14.3–97.2 kDa) and Premixed Protein Marker (High, 44.3–200.0 kDa) (Takara Co., Ltd., Dalian, China) were used to determine the molecular masses of proteins. The Premixed Protein Marker (3.4–100.0 kDa, Beijing Baoxidi Science and Technology Co., Ltd., Beijing, China) was used to determine the molecular mass of peptides. Samples of protein and peptide were mixed with 10 µL of electrophoresis buffer (0.5 M Tris-HCl, 10% SDS, glycerol, 0.5% bromophenol blue and 5% β-mercaptoethanol) and heated for 10 min at 100 °C. Tricine-SDS-PAGE gels (4% spacer gel and 16% separating gel) were used to resolve the peptide extracts. Proteins were separated by SDS-PAGE (5% spacer gel and 10% separating gel) [26,27]. The results were analyzed by Quantity One version 4.6.2 (Bio-Rad Technical Service Department, Oakland, CA, USA).

### 3.4. Activation of SPE Column

The SPE column was activated by the addition of 3 mL of methanol and equilibration with 3 mL of 0.1% (*v*/*v*) FA [28]. The peptides were then loaded onto the activated column and desalted with 1.5 mL of 0.1% FA (*v*/*v*). Finally, the peptides were eluted with 80% (*v*/*v*) methanol. Room temperature was operating condition. The eluted fractions were dried under a stream of nitrogen and dissolved in 0.1% FA (*v*/*v*) before detection by MS.

### 3.5. Peptide Identification by UPLC-Q-TOF

UPLC-Q-TOF method was developed to identify peptides from protein hydrolysates. The peptides were analyzed using MS/MS (Bruker Scientific Technology Co., Ltd., Beijing, China) with ESI ion source [28] coupled with an LC system (Thermo Fisher Scientific, California, USA) [29]. Peptide sequences were deduced based on MS/MS fragmentation data. The liquid phase-adopting gradient elution program was carried using a C18 column (Guangzhou Phenomenex science instrument Co., Ltd., Guangzhou, China). The program time was 70 min. The mobile phase was 0.1% FA in water (eluent A) and 0.1% FA in acetonitrile (eluent B). The flow rate was 0.3 mL/min. The organic phase was increased from 5% to 10% for 12 min and changed 35% for 35 min, then reached 50% and organic phase was increased from 50% to 85% using 2 min and 85% remained 8 min, then quickly returned to 5% using 2 min. The resulting files were searched using the Mascot search software as the following: (1) Protein database were set from National Center for Biotechnology Information (NCBI, https://www.ncbi.nlm.nih.gov/); (2) the enzyme was set as Trypsin; (3) the significance threshold was set up as *p* < 0.05. The peptides sequence was identified through database matching as well as the manual interpretation of its MS/MS spectrum. Peptides with ion scores more than the identity threshold (score > 35) were regarded as identity peptides. The MS spectra were acquired under the positive ESI conditions [30].

Swiss-Prot database (http://www.ncbi.nlm.nih.gov/protein/?term=Swiss-Port) and Bivalvia database (http://www.ncbi.nlm.nih.gov/protein/?term=Bivalvia) were searched using the MASCOT server (Peptide tol: ±10.0 ppm, MS/MS tol: ±0.03 Da; Defined by user: 35.0; http://192./68.1.6/mascot/). Peptides mass fingerprints and sequence queries were analyzed and MS/MS ion search was performed, using MASCOT [31].

### 3.6. In Silico Predictions of Peptide Toxicity, Allergic and Antithrombotic Activity

The potential character of the identified peptides from E1–E3 samples was analyzed using ToxinPred (http://www.imtech.res.in/raghava/toxinpred/) and AlgPred (http://crdd.osdd.net/raghava//algpred/) [32,33]. In addition, the antithrombotic activity of the peptides was assessed using molecular docking. Molecular docking of the estimated antithrombotic peptides was carried out using Discovery Studio 2017 software (Neo trident Technology Ltd., Beijing China) according to a method described by Tu et al. [34] with some modifications. The peptides were processed by this software whose structures were energy minimized using steepest decent and conjugate gradient techniques. The corresponding receptor protein was from PDB database (http://www.rcsb.org/pdb/home/home.do) and also treated by completing the missing amino acids, removing water molecules and so on. Docking was performed using CDOCKER docking tool of Discovery Studio software (Neo Trident Technology Ltd., Beijing, China). The best ranked docking pose of peptides in the active site of antithrombotic was obtained according to the score of binding-energy value [35].

### 3.7. Determination of Thrombin Inhibitory Activity

The antithrombotic activity of 2, 3 and 5 mg/mL E1–E3 were determined. A microplate reader was set at a wavelength of 405 nm, at 37 °C. The fibrinogen, thrombin and E1–E3 were all dissolved in 50 mM Tris-HCl buffer (pH 7.4) containing 0.154 mM NaCl. 0.1% fibrinogen solution (140 μL) and 40 μL sample solution with various concentrations were added into the plate wells, mixed and then the absorbance of the sample blank was measured. Next, 10μL thrombin solution (12 U/mL) was added and incubated in 37 °C. Finally, the absorbance of 405 was measured after 10 min [36,37,38]. The control treatment contained 40 μL of Tris-HCl buffer instead of the sample solution. Inhibition rates were calculated according to the following equation; *C*, *Cb*, *S*, *Sb* represent the absorbance of control, control blank, sample, sample blank, respectively.

(2) $$\mathrm{Inhibition}\;\mathrm{Rate}(\%)=\frac{\left(C-Cb)-(S-Sb\right)}{C-Cb}\times 100\%$$

### 3.8. Statistical Analysis

Values are expressed as the mean ± SD (*n* ≥ 3). Following the assessment of significant differences between samples by one-way analysis of variance (ANOVA), the level of significance was set at *p* < 0.05. All statistical tests were conducted using SPSS software 19.0 (SPSS Inc., Chicago, IL, USA).

## 4. Conclusions

Three *M. edulis* protein fractions and their enzymatic hydrolysis products were obtained and analyzed. The content of sarcoplasmic, myofibrillar and matrix proteins in *M. edulis* was different and the peptide compositions of three kinds of proteins were also different by Venn diagrams. Altogether, 387 peptides were identified in enzymatic hydrolysates of *M. edulis* protein; none of the peptides were toxic and allergic. E3 and E2 showed better activity than E1 and results showed that the antithrombotic activity of hydrolysates may be related to protein type, the DH and types and properties of peptides according to active mechanism. The enzymatic hydrolysates of *M. edulis* proteins can be exploited by the food industry to improve nutritional value as a functional component. On the other hand, ELEDSLDSER was screened as an antithrombotic peptide and the described optimized preparation of bioactive peptides from different *M. edulis* protein fractions comprised a good theoretical basis for development of *M. edulis* protein and may be applied to further the research into bioactive peptides from mollusks.

## Figures and Tables

**Table 1:** Peptides in enzymatic hydrolysates of P1–P3 *M. edulis* protein fractions.

Sequence	Source	–CDOCKER ENERGY	Toxin	Allergic
ELEDSLDSER	P2, P3	191.202	non	non
ITIQQELEDAR	P2	189.481	non	non
STELLIR	P2, P3	188.957	non	non
EKDEEIDSIR	P3	182.45	non	non
GDEELDSLIK	P2, P3	174.548	non	non
EQEAKAELQR	P3	173.992	non	non
ISMLEEDIMK	P1, P2, P3	169.018	non	non
LAITEVDLER	P3	168.65	non	non
QEELEEIRR	P2, P3	164.775	non	non
IEDDYNSLQK	P1, P2, P3	164.759	non	non
LEDELLTEK	P1	163.193	non	non
LEEAEAQALK	P2	163.148	non	non
KLAITEVDLER	P3	162.253	non	non
QEELEEIR	P2, P3	160.569	non	non
ELEDLSER	P1, P2, P3	160.178	non	non
ALADEGSDIK	P1	160.159	non	non
NSFVNDIFER	P3	158.605	non	non
EDSYEETIR	P2	157.024	non	non
DLDSDVSSTR	P2, P3	155.361	non	non
VSDLAEDMKR	P3	154.278	non	non
EIAEILYDR	P1, P2	153.187	non	non
VEFLDDSNR	P1, P2	153.163	non	non
WIAEEADK	P1, P2, P3	152.732	non	non
VSDLAEDMK	P3	152.661	non	non
EIVDLVLDR	P1, P2	152.039	non	non
LQSEVTEINR	P3	151.634	non	non
VISLTDDTSK	P1, P2	151.11	non	non
IDALEGSNSR	P2, P3	150.169	non	non

Peptides in hydrolysates of P1–P3 protein fractions from *M. edulis* were identified by UHPLC-Q-TOF and MS data were analyzed using MASCOT. Non means no toxicity or sensitivity.
